# Supplementation of Water Spinach (*Ipomoea aquatica*) on the utilization of *Mimosa pigra* and *Leucaena leucocephala* leaf for *in vitro* fermentation

**DOI:** 10.14202/vetworld.2023.215-221

**Published:** 2023-01-30

**Authors:** Channy Sambo, Sreychou Heng, Pisey Vong, Kuyhor Te, Sath Keo, Mom Seng, Samnang Ven

**Affiliations:** 1 Department of Animal Nutrition, Faculty of Animal Science, Royal University of Agriculture, Phnom Penh, Cambodia; 2 Department of Scientific Research, Directorate General of Higher Education, Ministry of Education, Youth and Sports, Phnom Penh, Cambodia; 3 Department of Veterinary Epidemiology and Public Health, Faculty of Veterinary Medicine, Royal University of Agriculture, Phnom Penh, Cambodia; 4 Royal University of Agriculture, P.O. Box: 2696, Phnom Penh, Cambodia

**Keywords:** *In vitro* fermentation, *Leucaena leucocephala* leaf, *Mimosa pigra*, Nutrient digestibility, Supplementation, Utilization, Water Spinach (*Ipomoea Aquatica*)

## Abstract

**Background and Aim:**

*Ipomoea aquatica* (Water Spinach) is the most potential for livestock growth performance, including chickens, pigs, cattle, and goats, especially in a tropical country like Cambodia. It is not only an alternative feed source but also one kind of supplemented feed for goat raising. Supplementation with Water Spinach in the utilization of low-quality tree foliage results in an increase in dry matter intake in goat production. This study aimed to identify the effectiveness of supplementation of Water Spinach in the utilization of *Mimosa pigra* and *Leucaena leucocephala* leaf in *in vitro* fermentation.

**Materials and Methods:**

The study was designed according to a 2 × 2 factorial arrangement in randomized design of seven treatments with different ratios consisted of different three types of dietary treatments, including *M. pigra*, *L. leucocephala*, and Water Spinach. The treatments were arranged according to a completely randomized design and were as follow: T1 = *M. pigra* leaf (100%); T2 = *L. leucocephala* leaf (100%); T3 = *M. pigra* leaf and *L. leucocephala* leaf (50% and 50%); T4 = *M. pigra* leaf and Water Spinach (99.5% and 0.5%); T5 = *L. leucocephala* leaf and Water Spinach (99.5% and 0.5%); T6 = *M. pigra* leaf and Water Spinach (99% and 1%); and T7 = *L. leucocephala* leaf and Water Spinach (99% and 1%). A total of 200 mg (dry matter) of dietary treatments were prepared in a 60 mL syringe. Each treatment was replicated 3 time. Gas recording of each treatment lasted for 3 days. *In vitro* was performed for 72 h, was followed by Makkar method. Gas production was recorded at 2, 4, 8, 12, 24, 36, 48, and 72 h of incubation by using strict anaerobic technique. A mixture of rumen fluid and dietary treatments were carried out under continuous flushing with CO_2_ in sharking incubator at 39°C. After incubating for 72 h, the ammonia concentration (NH_3_-N) was measured and recorded to identify pH, nutrient digestibility, and ammonia concentration (NH_3_-N).

**Results:**

Nutrient digestibility of the treatment with Water Spinach supplement in the utilization of *L. leucocephala* was obtained at a higher digestibility than treatment with *M. pigra* (p < 0.05). Gas production was different between groups (p < 0.05). Treatment with only *M. pigra* leaf had the highest gas production (A), while treatment with Water Spinach supplementation had the lowest gas production (A). At 0–24 h, the treatment with *L. leucocephala* leaf and Water Spinach 0.5% had the highest gas production, but after 24 h, *M. pigra* leaf and Water Spinach 1% and *L. leucocephala* leaf and Water Spinach 0.5% produced more gas compared to the other treatments (p < 0.05).

**Conclusion:**

The supplementation of Water Spinach 1% in treatment with *M. pigra* and *L. leucocephala* leaf resulted in increased degradability, gas production, and NH_3_–N concentration without a change in the pH value rumen condition. Based on these results, it is recommended that the level of Water Spinach supplementation should be 1% of dietary intake. Future studies should consider investigating the rumen ecology associated with Water Spinach supplementation. Feeding with Water Spinach remains a good supplement for ruminant performance; therefore, further studies should be conducted using Water Spinach in ruminant feeding in both metabolic and feeding trials.

## Introduction

Most grass feeding ruminants contain a high fiber level of more than 40% and a low protein level of <10%, resulting in limited growth performance during the dry season [1]. Varieties of feeding programs are important to provide an appropriate nutrient balance to achieve the nutrient requirement for ruminant maintenance, growth, and production of animals [2]. In Cambodia, the source of ruminant feeding is based on natural grasses that are readily available in the rural area, especially legume trees and shrub foliage such as *Mimosa* and *Leucaena*.

However, *Mimosa pigra* is affected by biodiversity, fishing, and crops [3]. Using leguminous leaves in livestock feed would prevent this plant from becoming a serious concern through the overgrowth in the environment and plays an important role in sustainable development [4]. *Mimosa pigra* has been known as a high crude protein source that contained in the leaf and it was also generally used as a feed source in swines, rabbits, and goats [5]. Legume trees like; *Leucaena leucocephala* were introduced as an animal feed supplement [6]. It is a digestible protein and was often recommended as ruminant feed; however, feeding with only *L. leucocephala* may not affect intake of dry matter (DM) and other nutrients, resulting in digestive problems in the ruminant animals [7]. These two legume trees are considered to be a source of protein consumption in ruminant, but it contains high levels of tannin, which results in low digestion in the ruminant and contributes to low gas production [8]. Water Spinach is known as vegetative forage, which contains an adequate protein level for animal requirements when combined with legume trees [1]. It is, therefore, hypothesized that the growth performance of goats fed leguminous tree species as a basal diet would be improved by supplementing it with Water Spinach. Thus, the supplementation of Water Spinach would be considered as a quality additive to improve livestock production while utilizing *M. pigra* and *L. leucocephala* leaves. The increase in growth rate was consistent with the additional level of Water Spinach and it maintained great DM digestibility [9]. However, there are limited available studies on using Water Spinach supplementation with legume trees as a basal diet in ruminants. Therefore, utilizing *M. pigra* and *L. leucocephala* leaves in different proportions with different levels of Water Spinach supplement would be a promising concept to investigate a novel digestibility system in goats. To conduct this experiment, *in vitro* fermentation is a convenient technique compared to other digestibility methods in ruminant experiments [10].

Therefore, this study aimed to investigate supplementation of Water Spinach on the utilization of *M. pigra* and *L. leucocephala* leaf on *in vitro* fermentation using beef cattle rumen fluid as rumen inoculum to evaluate ammonia concentration, pH, and nutrient digestibility in *in vitro* gas production methods.

## Materials and Methods

### Ethical approval

In this study, we did not use live animals; therefore, ethical approval is not required. Rumen fluid was obtained from two beef cattle sacrificed in the slaughterhouse.

### Study period and location

The study was conducted from July to August 2019 on beef cattle. Twenty days of adaptation period was performed in two animals with basal diet in individual pens of slaughterhouse area located in the north of Phnom Penh, before rumen fluid was collected. A total of 10 days included 1 day of rumen fluid collection in the slaughterhouse, 3 days of *in vitro* fermentation in laboratory, and 6 days of nutrient digestibility analysis. *In vitro* performance, and nutritional analysis was conducted in the Chemical Analysis Lab., Graduate school, Royal University of Agriculture, Dangkao district, Phnom Penh.

### Dietary substrate, treatments, and experimental design

The study designed was a 2 × 2 factorial arrangement in a randomized design of 7 treatments performed in triplicate, while the first factor was different levels of Water Spinach supplementation (0, 0.5, and 1% DM of the dietary substrate), and the second factor was tree forage utilization (non-supplemented, 50% of *Leucaena* and *Mimosa*, and 100% of *Leucaena* and *M. pigra* utilization DM of dietary substrate). The treatments were arranged according to a completely randomized design and were as follows: T1 = *M. pigra* leaf (100%); T2 = *L. leucocephala* leaf (100%); T3 = *M. pigra* leaf and *L. leucocephala* leaf (50% and 50%); T4 = *M. pigra* leaf and Water Spinach (99.5% and 0.5%); T5 = *L. leucocephala* leaf and Water Spinach (99.5% and 0.5%); T6 = *M. pigra* leaf and Water Spinach (99% and 1%); and T7 = *L. leucocephala* leaf and Water Spinach (99% and 1%). A total of 200 mg (DM) dietary treatments were prepared in a 60 mL syringe.

### Chemical composition analysis

To identify dietary chemical composition, substrates were milled to pass through a 1 mm screen (Cyclotec 1093 Sample mill, Tecator, Hoganas, Sweden), including 1–2 m lengths of *L. leucocephala* and *M. pigra* stems. The end of the stem was cut to measure about 30–50 cm long and was maintained for laboratory analysis [11].

### Medium solution preparation

A medium solution of 1000 mL was prepared for 33 plastic syringes that were obtained from the study conducted by Menke and Steingass [12]. Distilled water (475 mL), 240 mL macromineral solution (5.7 g Na_2_HPO_4_, 6.2 g KH_2_PO_4_, and 0.6 g MgSO_4_ made up to 1 L with distilled water), 240 mL buffer solution (35 g NaHCO_3_ and 0.4 g NH_4_HCO_3_ made up to 1 L with distilled water), 0.12 mL micromineral solution (13.2 g CaCl_2_H_2_O, 10 g MnCl_4_H_2_O, 1 g CoCl_2_6H_2_O, and 0.8 g FeCl_2_6H_2_O made up to 1 L with distilled water), 100 mg of resazurin, 2 mL of reducing buffer (1M NaOH, 336 mg Na_2_S.9H_2_O made up to 1 L with distilled water), and adding 47.5 mL distilled water to finalize the volume.

### Animals and preparation of rumen inoculums

Animals were placed on a routine feeding plan for at least 20 days and kept in individual pens with free access to clean, fresh water, and mineral blocks. On day 21, about 1500 mL of rumen fluid was obtained from the animals before feeding a morning mix of concentrate, grass, and rice straw while at the slaughterhouse. Both large and small ruminants have the same digestive system with a four-compartment stomach, The digestive systems have the same function that results from the same condition, such as pH level, ammonia concentrate, and nutrient digestibility in the rumen fluid [13]. The rumen fluid was transferred into prewarmed thermos-flasks and transported to the laboratory. The artificial saliva solution or buffer media preparation was prepared following that used in the study of Menke and Steingass [12]. A total of 1,500 mL rumen inoculum was prepared by the mixture of rumen fluid (500 mL) and artificial saliva 1,000 mL into 1.5 L thermos-flasks.

### *In vitro* fermentation of a substrate

About 200 mg (DM) of dietary treatments were weighed into 60 mL plastic syringes. The method used for *in vitro* fermentation was based on the technique described by Makkar *et al*. [14]. Strict anaerobic techniques were used in all steps during the rumen fluid transfer and incubation periods. The syringes with a mixture of substrate treatments were prewarmed in a water bath at 39°C for 1 h before filling with 30 mL of the rumen inoculum mixture [12]. Three blank syringes without samples but containing 30 mL of the medium containing the buffer and rumen fluid at a ratio of 2:1 were used as the control group.

### Gas production kinetics

Rumen fluid was collected from the slaughtered cattle at 4 am before feeding for the incubation solution and diluted at a (1:2) ratio (reducing medium: rumen fluid) in the collected artificial saliva in a CO_2_ tank. The 30 mL of rumen inoculum was added to each 60 mL plastic syringe with the dietary substrate (200 mg of DM). All syringes were incubated in a shaking water bath at 39°C for 72 h. During the incubation, the gas production kinetics were recorded at 0, 2, 4, 8, 12, 24, 36, 48, and 72 h. Cumulative gas production data were calculated based on the gas production at each time using the Fitcurve program fitted to the model of Orskov and Mcdonald [15] as follows: Y = A + B (1-e^(-ct)^), where A = The gas production from the immediately soluble fraction; B = The gas production from the insoluble fraction; C = The gas production rate constant for the insoluble fraction (B); t = Incubation time; (A + B) = The potential extent of gas production; and Y = Gas produced at a time “t”. After incubating for 72 h, the ammonia concentration (NH_3_–N) was measured and recorded to identify pH, nutrient digestibility, and ammonia concentration (NH_3_–N).

### Nutrient digestibility

To determine nutrient digestibility, the residue of *in vitro* fermentation was used to analyze the nutrient dry matter degradability (DMD) and organic matter degradability (OMD) based on the Association of Official Analytical Chemists [16].

### pH measurement

pH measurement was performed at 72 h of incubation using a pH meter (SM802, Romania). The pH meter is calibrated to pH buffers 7 and 4 before performing the pH measurement. Fermentation liquid was poured into a beaker and the probe was placed in a beaker to read and record the pH.

### Ammonia concentration (NH_3_–N)

Fermentation liquid was sampled at 18 h postinoculation and filtered through four layers of cheesecloth. Samples were kept in plastic bottles to which 2.5 mL of 1 M H_2_SO_4_ was added to stop the fermentation process of microbe activity and then centrifuged at 3,000× *g* for 10 min. The supernatant was stored at −20°C before the analysis of ammonia nitrogen (NH_3_–N) using the micro-Kjeldahl method [11].

### Statistical analysis

All obtained data were subjected to the General Linear Model (to analyze the interactions) procedures of the Statistical Analysis System Institute [17] through a 2 × 2 factorial arrangement in a randomized design of seven treatments performed in triplicate. The statistical model included *L. leucocephala* leaf: Water Spinach, and *M. pigra* leaf: Water Spinach interactions. Differences among the treatment means with p < 0.05 were contrasted using Tukey’s multiple comparison test [18].

## Results

### Chemical composition of dietary

The chemical compositions of the dietary feed are presented in Table-1. *Mimosa pigra* and *L. leucocephala* leaves contained CP at 15.6% and 24.5%, respectively. Comparing the CP contained in the three main dietary substrates, Water Spinach had a higher protein level than *M. pigra* and *L. leucocephala* leaves (Table-1).

**Table-1:** Nutrient composition of dietary feed (% DM).

Nutrient composition	Water Spinach	*M. pigra* leaf	*L. leucocephala* leaf
DM	10.0	39.6	24.9
OM	87.4	92.3	91.3
CP	30.0	15.6	24.5
NDF	26.2	32.9	25.1
ADF	24.8	27.9	15.9
CF	14.9	23.2	15.6
Ash	12.6	7.7	8.7

DM=Dry matter, OM=Organic matter, CP=Crude protein, NDF=Neutral detergent fiber, ADF=Acid detergent fiber, CF=Crude fiber, *L. leucocephala=Leucaena leucocephala*, *M. pigra*=*Mimosa pigra*, T_1_=*Mimosa pigra leaf*; T_2_=*L. leucocephala leaf*; T_3_=*Mimosa pigra* leaf + *L. leucocephala* leaf (50:50); T_4_=*Mimosa pigra* leaf + Water Spinach 0.5%; T_5_=*L. leucocephala* leaves + Water Spinach 0.5%; T_6_=*Mimosa pigra* leaf + Water Spinach 1%, and T_7_=*L. leucocephala* leaf + Water Spinach 1%

### pH and ammonia concentrations in the rumen fluid

Ruminal pH and NH_3_–N concentrate are presented in Table-2, which was not affected by the supplementation of Water Spinach and the pH range was within 6.77–6.87. In this study, the treatment of *L. leucocephala* leaf contained 44.9 mg/dL of NH_3_–N compared to *M. pigra* leaf, which contained 32.3 mg/dL.

**Table-2:** Nutritional digestibility and ammonia concentration in rumen.

Treatment	pH	NH3–N (mg/dL)	Digestibility (%)	

			DM	OM
T_1_	6.83	32.3^b^	41.5^b^	40.0^b^
T_2_	6.80	44.9^a^	57.8^a^	55.2^a^
T_3_	6.83	38.0^a,b^	54.8^a,b^	53.3^a^
T_4_	6.80	31.9^b^	53.1^a,b^	51.2^a^
T_5_	6.83	44.0^a^	57.5^a,b^	55.6^a^
T_6_	6.77	31.0^b^	53.9^a^	52.2^a^
T_7_	6.87	44.5*^a^*	59.3^a,b^	57.2^a^
SEM	0.016	1.557	1.623	1.257
p-value	0.815	0.003	0.034	0.000

T_1_=*Mimosa pigra* leaf; T_2_=*L. leucocephala* leaf; T_3_=*Mimosa pigra* leaf + *L. leucocephala* leaf (50:50); T_4_=*Mimosa pigra* leaf + Water Spinach 0.5%; T_5_=*L. leucocephala* leaves + Water Spinach 0.5%; T_6_=*Mimosa pigra* leaf + Water Spinach 1%, and T_7_=*L. leucocephala* leaf + Water Spinach 1%, SEM=Standard error of the mean, *L. leucocephala*=*Leucaena leucocephala*, *M. pigra*=*Mimosa pigra*. ^a,b,c^Superscripts differences in the same column show significant differences among. p *<* 0.05

### Gas production

Table-3 illustrates the values of A, B, A + B, and C of each dietary group and the statistically significant differences are indicated by p < 0.05. After 72 h of incubation with Water Spinach on the utilization of *M. pigra* and *L. leucocephala* leaves, there was an increase in gas production from the immediately soluble fraction (A), gas production from the insoluble fraction (B), gas potential extent of gas production (A + B), and cumulative gas production at 24 h, (p < 0.05) and it remained stable until 72 h (Table-3). A high gas production (mL/0.2 g DM) was found in the supplementation of Water Spinach with the utilization of *L. leucocephala* leaf (Table-3), which indicated that the treatment with *L. leucocephala* leaf + Water Spinach 0.5% gas production was higher than the treatment with *L. leucocephala* leaf + Water Spinach 1%.

**Table-3:** Supplementation of Water Spinach on the utilization of *M. pigra* leaf and *L. leucocephala* leaf on *in vitro* gas production.

Treatment	Gas production kinetics (mL/0.2 g DM substrate)				Gas 72 h (mL/0.2 g DM substrate)

	A	B	C	A+B	
T_1_	0.86a	26.5^d^	0.06c	27.4^c^	27.2^c^
T_2_	−0.07^a,b^	35.1^cd^	0.11^a,b^	35.0^b,c^	34.9b,^c^
T_3_	0.16^a,b^	33.5^c,d^	0.09^b^	33.7^b,c^	33.6^b,c^
T_4_	0.25^a,b^	26.6^b,c^	0.09^a,b^	26.8^b^	26.7^b^
T_5_	−1.01^b^	38.9^a,b^	0.11^a^	37.5^a,b^	37.5^a,b^
T_6_	−1.37^a,b^	25.9^a^	0.09^ab^	26.9^a^	27.7*^a^*
T_7_	−2.84^a,b^	38.1^a^	0.013^a,b^	37.0^a^	37.0^a^
SEM	0.316	1.38	0.004	1.214	1.219
p-value	0.039	0.020	0.006	0.006	0.005

A=The gas production from the immediately soluble fraction, B=The gas production from the insoluble fraction, C=The gas production rate constant for the insoluble fraction (B), A + B=The gas potential extent of gas production, *L. leucocephala*=*Leucaena leucocephala*, *M. pigra*=*Mimosa pigra*, SEM=Standard error of the mean, T_1_=*Mimosa pigra* leaf; T_2_=*L. leucocephala* leaf; T_3_=*Mimosa pigra* leaf + *L. leucocephala* leaf (50:50); T_4_=*Mimosa pigra* leaf + Water Spinach 0.5%; T_5_=*L. leucocephala* leaves + Water Spinach 0.5%; T_6_=*Mimosa pigra* leaf + Water Spinach 1%, and T_7_=*L. leucocephala* leaf + Water Spinach 1%. ^a,b,c^Superscripts differences in the same column show significant differences among. p *<* 0.05 treatment

### Nutrient digestibility

The effect of Water Spinach supplementation on pH value, ammonia concentration, and digestibility of each feed group are presented in Table-2. The addition of Water Spinach did not change the pH value (p = 0.815). The range of pH values is between 6.77 and 6.88. The group with *M. pigra* leaf had the lowest ammonia levels (p < 0.05), however, ammonia concentration significantly increased with Water Spinach supplementation (p = 0.003). Supplementation with 1% of Water Spinach using *L. leucocephala* and *M. pigra* as basal diet resulted in better digestion compared to the 0.5% supplemented and control groups. In addition, DMD and OMD from the treatment group with *L. leucocephala* leaf with Water Spinach were higher than the treatment group with *M. pigra* leaf. A high DM digestibility was present in the group supplemented with 1% of Water Spinach (T_7_) which accounted for 59.3% compared to T_4_ (53.1%) and T_5_ (57.5%).

## Discussion

This study aimed to investigate the supplementation of Water Spinach on the utilization of *M. pigra* and *L. leucocephala* leaf on *in vitro* fermentation using beef cattle rumen fluid as rumen inoculum to evaluate ammonia concentration, pH, and nutrient digestibility in *in vitro* gas production methods. The findings of this study are similar to the study of Wittayakun *et al*. [19], who reported that DM, CP, and OM content of *M. pigra* was 40.22%, 18.11%, and 92.93%, respectively. However, the *M. pigra* leaf chemical composition in this study had a low neutral detergent fiber (NDF) (32.9%) and acid detergent fiber (ADF) (27.9%) compared to values obtained by Wittayakun *et al*. [19], who reported that the NDF and ADF content of *M. pigra* was 54.7% and 39.9%, respectively. The chemical composition of Water Spinach in this study found that CP and crude fiber (CF) were 30.0% and 14.9%. In contrast, a previous study demonstrated that CP and NDF content was 19.5% and 41.5%, respectively [19]. According to the research by Hasanah *et al*. [20] and Opene *et al*. [21], Water Spinach contained CP levels between 10.65%–24.6%, 13%–21.62% of CFs, 10.3% DM, and 89.42% OM. Some reports have varying results, which may be due to different varieties, periods of sample collection, and soil types. In this study, *L. leucocephala* leaf contained 24.5% of CP, while the study by Makmur *et al*. [22] reported that *L. leucocephala* contained 21%–25% of CP. This result is similar to this study. It may be because of the dietary substrate sampling method; the previous study by Makmur *et al*. [22] collected all parts of plants, such as the leaves, flowers, and soft stems to investigate the chemical composition of each sample. However, this experiment used only the leaves of *L. leucocephala*.

To respond to the microbial efficiency in ruminants, the pH measurements in this study were within the normal range (6.77–6.87) compared to those reported by Truong and Van Thu [23]. Therefore, this supplementation of Water Spinach did not affect the ruminal pH (p = 0.81) (Table-2). On the other hand, this pH result did not significantly decrease because the dietary substrates were not fermentable carbohydrates [24]. This is similar to the results reported by Viennasay and Wanapat [25], which showed that the pH value was high (6.80–6.92) when feeding with concentrated feed.

With regard to the report of Rigueira *et al*. [26], NH_3_–N ranged from 3.3 to 4.4 mg/dL can be changed by the type or quality of feeding. The findings of this study are in accordance with the study of Lanyasunya *et al*. [27], which demonstrated increased NH_3_–N levels, the increase in NH_3_–N was 66.3–104.7 mg/L in cows fed with a large variety of plant forage. In this study, the treatments with Water Spinach supplement presented a significantly higher NH_3_–N concentrate than treatments without the Water Spinach supplement (Table-2). This increase may be due to Water Spinach being easily broken down by microorganisms.

The results of gas production are in accordance with the study of Emmanuel *et al*. [28], which reported that Water Spinach produced more gas after utilization with *M. pigra* and *L. leucocephala* leaves. While comparing with or without Water Spinach supplementation, gas production from *M. pigra* and *L. leucocephala* was reported to be 27.2 mL/0.2 g DM and 34.9 mL/0.2 g DM, respectively. This result was higher compared to the study by Malik *et al*. [29], who indicated that gas production from *M. pudica* utilization was about 24.4 mL/0.2 g DM. This may be because of different varieties of *Mimosa*, which originate from different locations.

The total gas production increased considerably by 20% when Water Spinach was used as a substitute for forage source in ruminants [30]. On the other hand, a previous study confirmed that supplementation with Water Spinach feed resulted in high gas production in ruminants and an increase in the digestibility and N retention of foliage in small ruminants, which could contribute to the high gas production [9]. This may contribute to the negative impact on protein and energy metabolism when goats are fed with a high level of dietary Water Spinach [9]. Hasanah *et al*. [20] found that the increase in gas production was shown in the low percentage of supplementation compared to the high concentration of supplement, which is similar to this study results. This study found that there was a high gas production in the utilization of *L. leucocephala* supplement with 0.5% of Water Spinach. With regards to the result reported by Soto *et al*. [31], gas production ranged between 34.4 and 37.6 mL/0.2 g DM for 24 h of incubation with vegetable waste as the diet. This may be because the large forage diet digestibility of each substrate may result in increased gas production [32]. On the other hand, the low content of lignin in Water Spinach may result in increased digestion in ruminants [20]. Using only legume trees in ruminant feed may contribute to low gas production (Table-3); this may be because of the high lignin content in *M. pigra* and *L. leucocephala*. In contrast, the gas production shown in Table-3 was considerably increased when there was supplementation with Water Spinach. A high amount of gas production was shown in the utilization of *L. leucocephala* compared to *M. pigra* by supplementation of Water Spinach (*Ipomoea Aquatica*). This is because of the high CP and NDF content of *L. leucocephala*, which results in high gas production (Table-3).

Nutritional digestibility is shown in Table-2. The DMD and OMD from the treatment with *L. leucocephala* leaf with Water Spinach were higher than the treatment with *M. pigra* leaf. This may be due to the high level of NDF and ADF in forage diets which affect the ruminal pH and result in limited rumen microbial growth, which affects intestinal digestibility [20]. In addition, DM and OM digestibility is presented in Table-2, which illustrates that DM digestibility in the treatment with supplementation of 1% Water Spinach in *L. leucocephala* was 59.3% (T7) compared to the treatment (T6) with *M. pigra* (53.9%), and OM digestibility was 57.2% (T7) and 52.2% (T6). These can be contributed that the 1% supplementation of Water Spinach in *L. leucocephala* usage, which resulted in more than 5% DM digestibility compared to the utilization of *M. pigra*.

This result seems to be consistent with the study by Wittayakun *et al*. [19], which showed that DM digestibility of *M. pigra* (giant sensitive tree) leaves and leaves with rachis was only 46.74% and 40.81%, respectively, and the OM digestibility was about 42.17% and 40.63%, respectively. This previous result was low compared to this study; it is possibly because there was no supplementation in feeding method. Adding Water Spinach in the utilization of *M*. *pigra* and *L. leucocephala* may provide a protein source for cellulolytic bacteria that grow in the digestive system; they also play an important role in the breakdown of other nutrients [33]. This possibly contributes to the great nutrient digestibility in the rumen when there was a Water Spinach supplement.

## Conclusion

This study on the supplementation of Water Spinach with the utilization of *M. pigra* and *L. leucocephala* leaves on *in vitro* fermentation has shown that *M. pigra* and *L. leucocephala* leaves are plants that are rich in protein, which is a microbial requirement for growth, digestion, and fermentation. These supplements have proven to be recyclable food supplements for ruminants to ensure a balance based on nutrient values and can increase ammonia levels without causing a decrease in pH value. Moreover, a 0.5% Water Spinach supplementation of the dietary substrate could be sufficient for ruminant growth and performance when legume forage is used as a basal diet. According to the results of this experiment, it may contribute data for further studies to investigate the growth rate and methane production from the supplementation of Water Spinach in rumen digestibility.

## Authors’ Contributions

SK, KT, MS, PV, and SV: Supervised the study. CS: Conducted the experiment. SV: Drafted the manuscript. SK: Designed the study. KT: Collected the data. MS, PV, and SH: Data analysis All authors have read and approved the final manuscript.
